# Study on Mechanical Properties of Carbon Nanotube Reinforced Composites

**DOI:** 10.3390/polym15163362

**Published:** 2023-08-10

**Authors:** Zhouyi Li, Haoran Liu, Yuan Li

**Affiliations:** 1 School of Aeronautics, Northwestern Polytechnical University, Xi’an 710072, China; lizhouyi1993@163.com; 2 School of Civil Engineering and Architecture, Xi’an University of Technology, Xi’an 710048, China

**Keywords:** composite structures, carbon nanotube, interlaminar toughening, high strain rate

## Abstract

In this study, carbon fiber composite laminates were modified by carbon nanotube films. In-plane and out-of-plane compression tests were carried out in a wide strain rate range (10^−3^–10^3^/s). Results display that the out-of-plane compressive properties are improved by CNT interlaminar toughening because CNT can hinder the propagation of interlayer cracks; however, the dynamic in-plane compression performance is decreased due to the lack of resin in CNT film that leads to delamination inside of CNT film in advance. To optimize the material preparation process, two methods were used to prepare the mode I fracture test: (a) curing the prepreg by autoclave process; and (b) curing of resin preform by vacuum resin-transmitted molding (VARTM). Results showed that CNT prolonged the crack propagation path and improved the interlaminar fracture properties when the preform was infiltrated with resin and cured by VARTM. In addition, it was found that the interlaminar thickness was almost linear with the number of CNT layers.

## 1. Introduction

Carbon fiber composites have developed rapidly in recent decades and are widely used in aerospace, automobile, architecture and other fields. Compared with traditional materials, such as aluminum alloy and steel, carbon fiber reinforced resin matrix composites have the characteristics of high specific strength, high specific stiffness and good fatigue resistance [1]. But there exist some disadvantages of composite laminates, such as low interlaminar toughness and insufficient resistance to impact damage. They are prone to delaminate especially when subjected to low-energy impacts. Therefore, some interlaminar toughening methods are needed to improve the interlaminar toughness. The main toughening methods include particle toughening [2], film toughening [3] and fiber toughening [4], which should ensure the light weight of the composite material. In recent decades, the development of nanocomposites represented by carbon nanotubes, nanoparticles, graphene and so on has advanced rapidly. Carbon nanotubes (CNTs) were first discovered in 1991 [5]. Their elastic modulus can reach 1.34 TPa and strength can reach 200 GPa. At the same time, CNTs have the advantages of large aspect ratio, high specific surface area and excellent electrical properties, which make them an ideal material for reinforced toughening and functionalization of composites [6].

Some researchers have prepared hybrid composites by mixing CNTs, as toughening phases, with fibers and resins to improve interlaminate properties to varying degrees. Experimental results showed that by adding double-walled carbon nanotubes with a mass fraction of 0.3% to the glass fiber interlayer and preparing laminates using a resin introduction molding process, the interlaminar shear strength increased by about 20% [7]. The reason for this increase was that the strength of the resin and the fiber-resin interface were both improved after the addition of carbon nanotubes. Hsiao [8] conducted shear tests on multi-walled carbon nanotube-modified carbon fiber resin matrix composites and found that the shear strength of the material increased by 46% with the addition of 5% carbon nanotubes. Karapappas et al. [9] made prepreg films by dispersing multi-walled carbon nanotubes in epoxy resin. Then they inserted the films in the form of interlayers between carbon fiber layers and cured to obtain hybrid composites. They found that the laminate G_IC_ increased by 60% and G_IIC_ increased by 75% at 1% CNT content. Romhany et al. [10] added MWCNT to the resin blend and found the highest improvement of carbon fiber composites was only 33% for CNT content of 0.3%. However, as the content of carbon nanotubes continued to increase, the viscosity of the resin increased dramatically and the CNTs agglomerated, causing difficulties in resin infusion and pores inside the formed material, resulting in the degradation of the material properties. In order to solve the problem of difficult dispersion of CNTs caused by the conventional process, researchers have proposed new solutions. Boroujeni et al. [11] prepared composites by growing carbon nanotubes directly on the surface of carbon fibers, and the results showed that their axial tensile strength and toughness were increased by 11% and 35%, respectively. Garcia et al. [12] prepared vertically grown CNT arrays at a high temperature and transferred them to carbon fiber prepreg by “transfer-printing” at room temperature, maintaining the orientation of carbon nanotubes in the thickness direction, and hot pressing the composite laminates, which increased the GI_C_ by 1.5–2.5 times and GII_C_ by 3 times. This indicates that carbon nanotubes can effectively improve the ability of composite materials to resist crack expansion. Rathi et al. [13] decorated the surfaces of multi-walled CNTs by zirconium dioxide (ZrO_2_) nanoparticles and synthesized MWCNT/ZrO_2_-based hybrid epoxy nanocomposites. The results reveal that the fracture toughness of hybrid composites is improved by ~31% compared to the neat epoxy when 1.0 wt% loading of nanofillers is used.

Moreover, researchers proposed a novel method, which by carbon nanotube films were inserted as an interlayer to achieve interlaminar toughening [14,15,16]. The CNT films were prepared by the floating catalyst chemical vapor deposition (FCCVD) method [17], in which the internal CNTs are cross-linked to form a network with a self-supporting structure and can be obtained by controlling the deposition time of CNTs with different surface densities of aggregates. The CNT films can be prepared by a simple process that avoids the above problems while increasing the CNT content and can be directly used in the preparation of composites [18]. After adding CNT film in the interlayer, the bending and shear strength first rise but eventually drop with the increase of CNT layers [19].

In this study, CNT films prepared by the FCCVD method were used for interlaminar toughening of carbon fiber resin matrix composites. First, quasi-static and dynamic in-plane and out-of-plane compression tests were conducted to analyze the failure modes of the materials and the effect of CNT films on the compression properties. Then, the quasi-static mode I interlayer fracture behaviors of the prepared materials with two curing processes (hot press and vacuum-assisted resin molding) were compared, and the crack expansion paths were analyzed to optimize the material preparation method. Finally, the interlayer toughened materials with different number of layers of CNT films were prepared, and the relationship between the number of CNT layers and the thickness of the interlayer was determined to support the subsequent research.

## 2. Sample Preparation and Test Method

### 2.1. Sample Preparation

**Figure 1:**
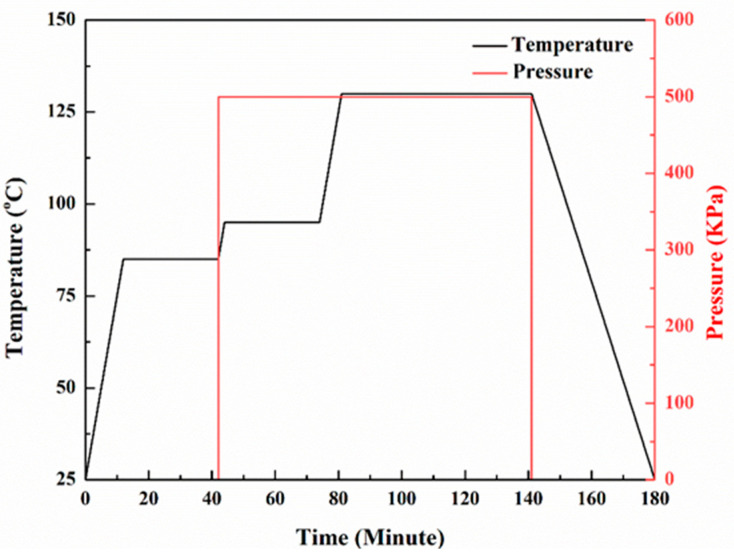
Curing process of autoclave.

**Figure 2:**
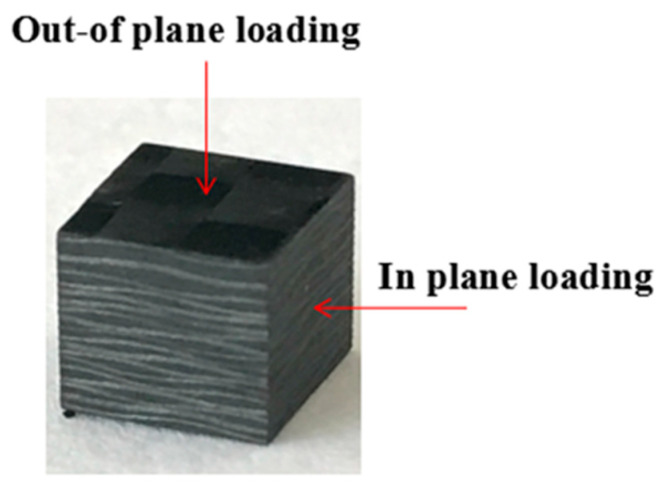
Compression sample.

The carbon fiber prepreg is used as the raw material and solidified using the autoclave process to prepare the sample required for compression testing. A total of 20 layers of two-dimensional woven carbon fiber resin-based prepreg (T700, 12 K) are laid. For toughened samples, two layers of CNT films (0.6 g/m^2^, Suzhou Jiedi Nano Technology Co., Ltd., Suzhou, China) are laid and deposited between each adjacent two layers of carbon fiber prepreg. The curing process is shown in Figure 1. After the curing is completed, the laminate is placed at room temperature, then is removed and cut into small cubic specimens of 5 × 5 × 5 mm^3^, and the specimens are shown in Figure 2.

**Figure 3:**
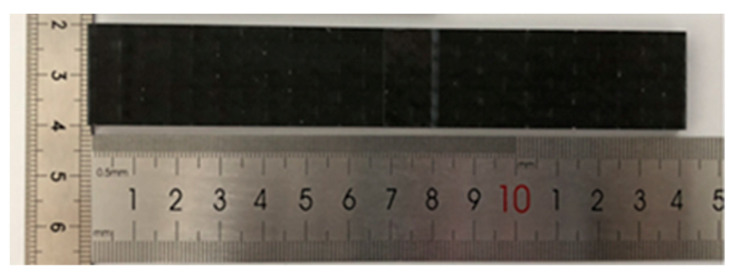
DCB sample.

Mode I fracture specimens are prepared using unidirectional carbon fiber prepreg and unidirectional carbon fiber dry cloth as raw materials, respectively, with carbon fiber type T300, 12 K. The resin type infused into the carbon fiber dry cloth is Araldite LY 1564 SP CIN, and the curing agent used is Aradur 3486 Blue CI (Hunstman, Shanghai, China) with a mass ratio of 10:3. To cure the carbon fiber prepreg, a hot pressing method was utilized by an autoclave. For carbon fiber dry cloth, curing is performed by the vacuum-assisted resin molding process (VARTM), which is carried out in an oven at 100 °C for 4 h and then cooled to room temperature. Teflon film with a thickness of 20 μm and a length of 80 mm is used as a pre-crack between the two middle layers of carbon fibers. For toughened materials, CNT films are then placed between the middle two carbon fiber layers. After the laminate is prepared, the material is cut using a diamond grinding wheel and a diamond wire saw. The spacing between the specimen and the laminate is kept at 30 mm around the cutting, and the mode I fracture specimen obtained is shown in Figure 3.

**Figure 4:**
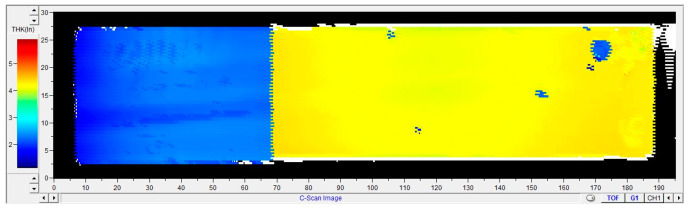
C-scan results of sample.

C-scan is performed on the prepared specimens to observe whether there are defects inside the specimens. The results are shown in Figure 4, which shows that there are basically no defects inside the specimens.

### 2.2. Test Method

#### 2.2.1. Test Method for Mechanical Properties of Compression

The quasi-static compression mechanical property testing equipment is an electronic universal testing machine (10 KN). The loading displacement of the test machine is controlled so that the loading strain rate is 10^−3^/s, and at least three repetitions of each condition are performed to ensure the validity of the data.

**Figure 5:**
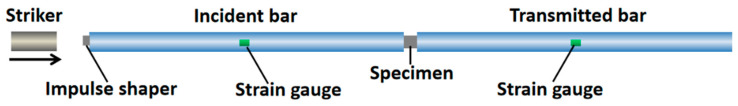
Schematic diagram of split Hopkinson pressure bar.

The dynamic compression test is carried out with a split Hopkinson pressure bar, and the schematic diagram of the device is shown in Figure 5. The strain rate range of the split Hopkinson bar is generally 10^2^–10^4^/s. The principle of operation is that the impact bar is controlled by air pressure to hit one end of the incident bar resulting in a compression wave in the incident bar, which propagates forward along the incident bar. When the compression wave is transmitted to the contact surface between the incident bar and the sample, one part is reflected at the free end of the incident bar, and the other part acts on the sample and then propagates to the transmitted bar. The incident wave can be measured by the strain gauges attached to the middle of the rod $\varepsilon_{inc}$, reflected wave $\varepsilon_{ref}$ and transmitted wave $\varepsilon_{tran}$. Using the one-dimensional stress wave theory [20], the strain rate of the sample is obtained $\dot{\varepsilon_{s}}\left(t\right)$, strain $\varepsilon_{s}\left(t\right)$, and stress $\sigma_{s}\left(t\right)$, as shown in Formulas (1)–(3).

(1) $$\;\;\;\;\;\;\;\;\;\;\;\;\;\;\;\;\;\;\;\;\;\;\;\;\;\;\;\;\;\;\;\;\;\;\;\;\;\;\;\;\;\;\;\;\;\;\;\;\dot{\varepsilon_{s}}\left(t\right)=\frac{2C_{0}}{L}\varepsilon_{ref}\left(t\right)\;\;\;\;\;\;\;\;\;\;\;\;\;\;\;\;\;\;\;\;\;\;\;\;\;\;\;\;\;\;\;\;\;\;\;\;\;\;\;\;\;\;\;\;\;\;\;$$

(2) $$\;\;\;\;\;\;\;\;\;\;\;\;\;\;\;\;\;\;\;\;\;\;\;\;\;\;\;\;\;\;\;\;\;\;\;\;\;\;\;\;\;\;\;\;\;\;\varepsilon_{s}\left(t\right)=\frac{2C_{0}}{L}\int_{0}^{t}\varepsilon_{ref}\left(t\right)dt\;\;\;\;\;\;\;\;\;\;\;\;\;\;\;\;\;\;\;\;\;\;\;\;\;\;\;\;\;\;\;\;\;\;\;\;\;\;\;$$

(3) $$\;\;\;\;\;\;\;\;\;\;\;\;\;\;\;\;\;\;\;\;\;\;\;\;\;\;\;\;\;\;\;\;\;\;\;\;\;\;\;\;\;\;\;\;\;\;\;\;\;\sigma_{s}\left(t\right)=E\left(\frac{A}{A_{s}}\right)\varepsilon_{tran}\left(t\right)\;\;\;\;\;\;\;\;\;\;\;\;\;\;\;\;\;\;\;\;\;\;\;\;\;\;\;\;\;\;\;\;\;\;\;\;\;\;\;$$

where L is the length of the specimen. E and $\;C_{0}$ are the modulus of elasticity and elastic wave velocity of the incident rod, respectively. A and $\;A_{s}$ are the cross-sectional areas of the incident rod and the specimen, respectively. A high-speed camera (Phantom v711, Vision Research Ltd., Wayne, NJ, USA) is used to take pictures of the loading process during the dynamic test to observe the deformation process at a frame rate of 200,000 fps.

#### 2.2.2. Interlaminar Mode I Fracture Test Method

**Figure 6:**
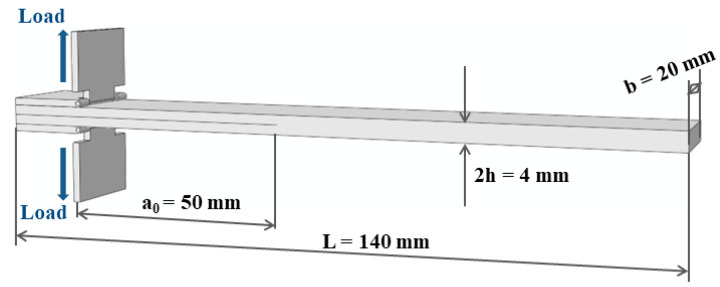
Loading mode and sample size of double cantilever beam test.

According to ASTM-D5528 test standard [21], double cantilever beam (DCB) specimens are used for the test. The thickness of the specimens should be 3–5 mm, and the thickness variation between each specimen should not exceed 0.1 mm. The quasi-static mode I interlaminar fracture loading mode and sample size are shown in Figure 6.

**Figure 7:**
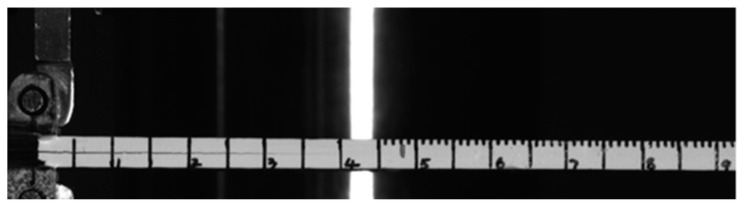
Process of mode I interlaminar fracture test.

The quasi-static mode I fracture test is conducted on the Instron 5848 electronic universal testing machine (Norwood, MA, USA) with a 100 N sensor. Before the test, one side of the specimen is painted and scaled using white correction fluid to facilitate the observation of the crack length during the test, as shown in Figure 7. The speed of the test machine is controlled so that the test loading speed is 1 mm/min and 10 mm/min. The test process is photographed using an industrial camera (JAI-sp-2000M, JAI, Copenhagen, Denmark). The camera is triggered at the same time as the test starts loading, which facilitates accurate recording of the load and displacement corresponding to the crack extension process. In order to avoid the influence of the possible resin-rich zone at the tip of the precast crack on the test, a preload is performed first. Then, the crack is unloaded at 3–5 mm of crack expansion and the front end of the precast crack is re-marked. This is followed by a second loading at the same rate until the specimen is completely fractured between the layers. To ensure the reliability of the data, the test is repeated at least three times for each working condition.

The energy release rate is calculated using the MBT method (modified beam theory) with the following equation [21]:

(4) $$\;\;\;\;\;\;\;\;\;\;\;\;\;\;\;\;\;\;\;\;\;\;\;\;\;\;\;\;\;\;\;\;\;\;\;\;\;\;\;\;\;\;\;\;\;\;\;\;\;\;\;\;G_{IC}=\frac{3P\delta }{2b\left(a+|\Delta |\right)}\;\;\;\;\;\;\;\;\;\;\;\;\;\;\;\;\;\;\;\;\;\;\;\;\;\;\;\;\;\;\;\;\;\;\;\;\;\;\;\;\;\;\;\;\;\;\;\;\;\;\;\;\;\;\;$$

where P is the external load, δ is the displacement of the loading point, b is the width of the specimen, a is the crack growth length. Δ is determined by making a straight line of the cube root of the flexibility (δ/P) and the primary function of the crack length by the least square method, and the horizontal coordinate of its intersection with the *x*-axis is Δ.

The method with a 5% decrease in stiffness (the slope of the load-displacement curve with a 5% decrease in stiffness is used as a straight line, and the intersection point with the load-displacement curve is taken as the crack initiation point; however, if the intersection point is after the maximum load, the point of maximum load is taken as the cracking point) is chosen to determine the crack initiation point. In addition, the corresponding energy release rate is the fracture toughness ($G_{Ic}$).

## 3. Test Results and Discussion

### 3.1. Quasi Static and Dynamic Compression Performance

**Figure 8:**
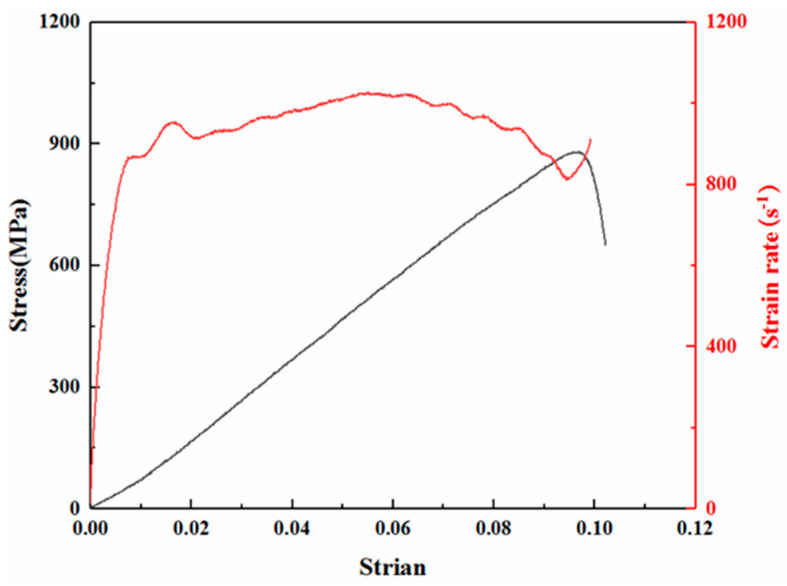
Typical stress–strain curve and strain rate history curve under dynamic loading.

The specimens prepared using the carbon fiber prepreg are first tested in compression in the in-plane and out-of-plane directions with the loading method shown in Figure 2. The incident wave shaping under the dynamics makes the stress inside the specimen balanced. The typical stress–strain curve is shown in Figure 8. The carbon fiber resin-based composite is referred to as CF/EP and the CNT interlayer-modified composite is referred to as CNT/CF/EP. The test results are shown in Table 1. The energy consumption refers to the area surrounded by the stress–strain curve.

From the experimental results, the out-of-plane compression performance of the CNT film-modified material is improved compared with the unmodified material, but the in-plane compression performance increases very little or even decreases slightly with the increase of strain rate.

**Figure 9:**
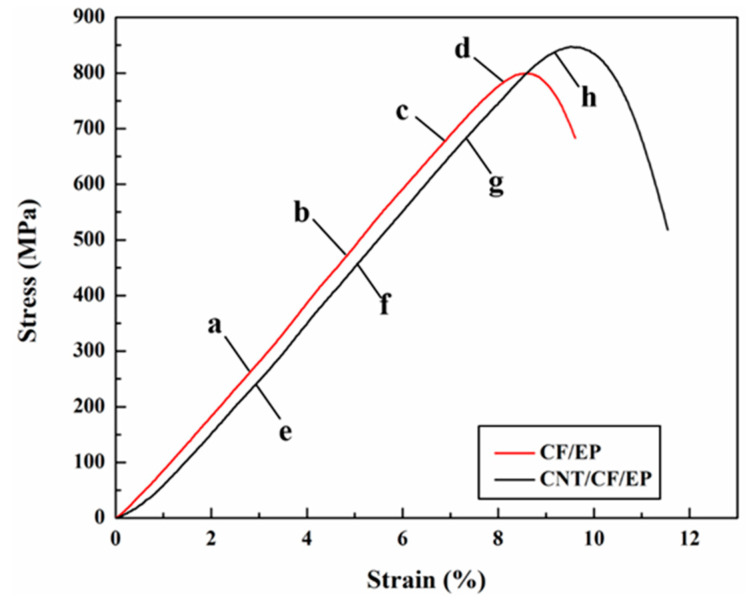
Stress strain curve of out-of-plane compression with 2500/s.

**Figure 10:**
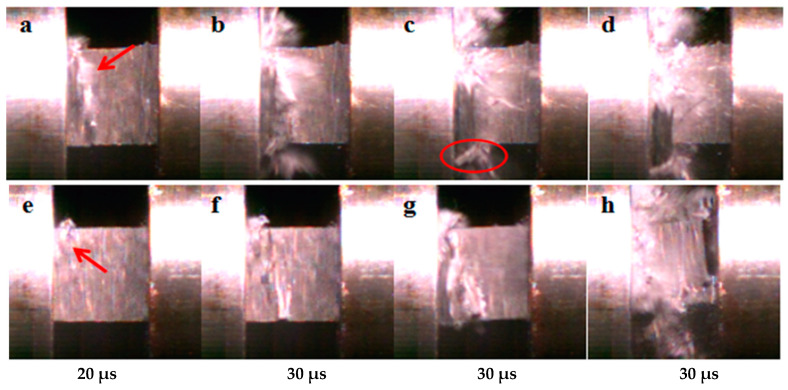
Failure process of out-of-plane dynamic compression specimen (**a**–**d**) CF/EP; (**e**–**h**) CNT/CF/EP.

The stress–strain curves under out-of-plane loading with a strain rate of 2500/s are shown in Figure 9, where a–d represent the state of CF/EP at different times and e–h represent the state of CNT/CF/EP at the same time. The compressive strength and energy absorption are improved by adding CNT films. The main damage mode of the composite under out-of-plane compression loading is matrix fracture, and the nonlinear segments in the stress–strain curves represent matrix cracking and fiber–matrix debonding. Figure 10 shows the deformation and damage process of the specimen taken using a high-speed camera. From Figure 10a pointed by the red arrow, it can be seen that significant cracking occurred in the CF/EP specimen interlayer resin, while no significant cracking occurred in the CNT/CF/EP interlayer resin (Figure 10e pointed by the red arrow). As the stress level in the specimens increased, larger damage areas were generated inside the CF/EP, while the fibers split and the specimens were continuously crushed (shown in the red circle in Figure 10c). For CNT/CF/EP, it can be seen from Figure 10f,g that no large-scale cracks were generated in the specimens. The CNT film with continuous network structure makes the composite layers more tightly connected, which effectively hinders the crack expansion and delays the specimens from being crushed. Thus, the compression strength and energy absorption of the material are effectively improved.

**Figure 11:**
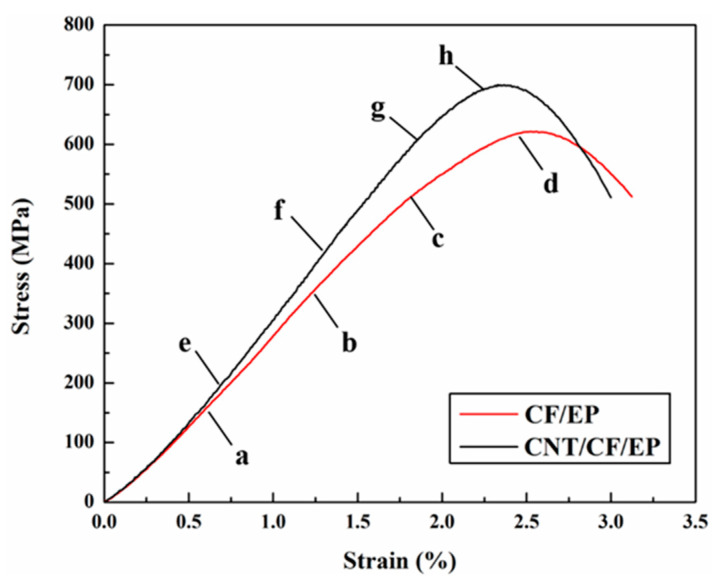
Stress strain curve of in-plane compression with 600/s.

**Figure 12:**
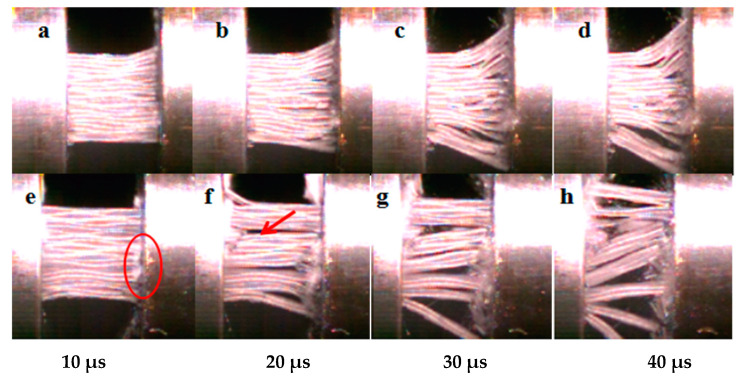
In-plane dynamic compression specimen failure process: (**a**–**d**) CF/EP; (**e**–**h**) CNT/CF/EP.

The stress–strain curve under in-plane loading at 600/s is shown in Figure 11, in which a–d represent the state of CF/EP at different times and e-h represent the state of CNT/CF/EP. The addition of CNT film material increases the compressive strength by about 10%, but the energy absorption remains almost unchanged due to the decrease in the corresponding failure strain. The results from the high-speed camera are shown in Figure 12. At 10 μs, the fibers of CF/EP buckled, while the fibers in CNT/CF/EP did not buckle significantly (as shown by the red circle in Figure 12e). This indicates a high load-bearing capacity without delamination, suggesting that the CNT film connects the layers well and effectively delays the generation of cracks in the resin zone between the layers. However, at 20 μs, CNT/CF/EP delamination is more obvious (pointed by the red arrow in Figure 12f) while the fibers are crushed; further fiber buckling is seen in CF/EP (Figure 12b). At 40 μs, the material approaches the stress peak and the CNT/CF/EP interlayer is basically completely separated and the fibers are crushed. Fiber buckling deformation is obvious but delamination is lower in CF/EP. Comparing the failure modes of the two materials, CF/EP has a high failure strain because its main damage mode is fiber buckling. Because it is mainly fiber crushing and delamination of the material, CNT/CF/EP has a high compressive strength but low failure strain.

**Figure 13:**
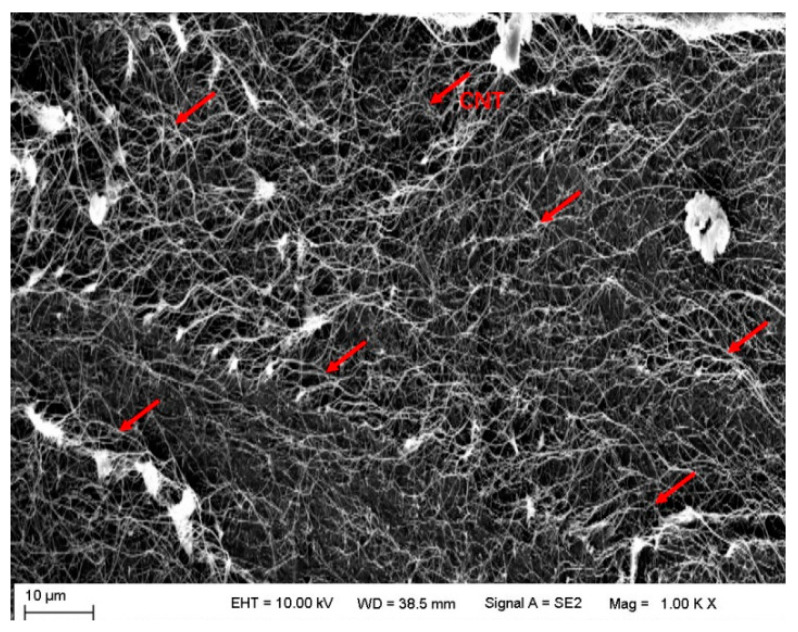
Microscopic image of fracture surface of CNT film interlayer-modified sample after in-plane compression failure.

In order to analyze the cause of delamination of the CNT/CF/EP sample, all fractures of the sample are observed by scanning electron microscope, and the results are shown in Figure 13. It can be seen that there is continuous CNT distribution on all fractures and almost no resin particles, which indicates that the delamination occurs inside the film due to insufficient resin infiltration in the CNT film. Due to the limited resin content in the carbon fiber prepreg, insufficient film wetting resulted in severe delamination during in-plane compression.

### 3.2. Mode I Interlaminar Fracture Behavior under Quasi-Static Loading 

**Figure 14:**
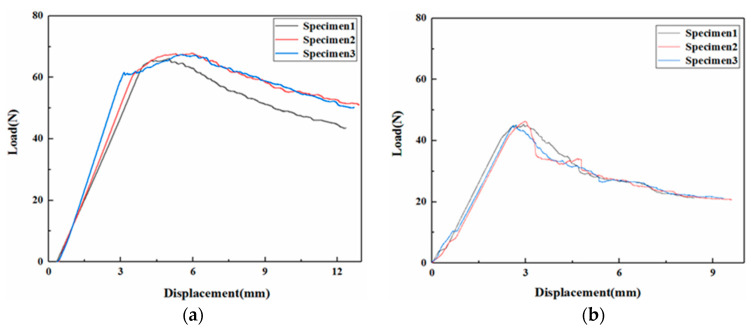
Mode I test’s load-displacement curve of specimens cured by hot press. (**a**) Untoughened sample. (**b**) CNT film toughened sample.

**Figure 15:**
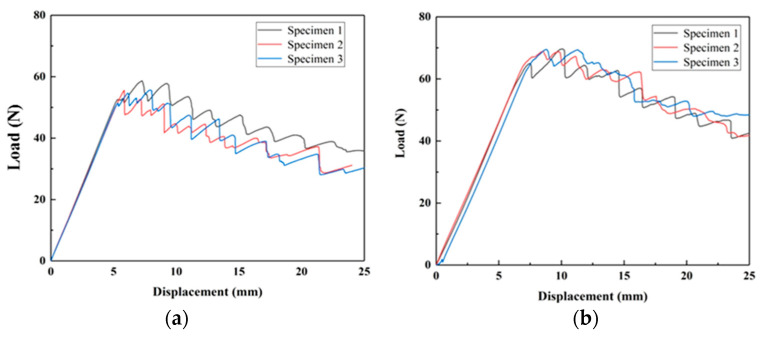
Mode I test load-displacement curve of specimens cured by VARTM. (**a**) Untoughened sample. (**b**) CNT film-toughened sample.

The materials prepared by the two curing processes are tested for tension-driven open fracture (mode I) under quasi-static conditions and their fracture properties are compared. Figure 14 shows the load-displacement curves of the carbon fiber prepreg cured and molded materials by hot press cans. Figure 14a shows the test results for the untoughened specimen. It can be seen that the force at the end of the linear section is about 62 N, corresponding to a displacement of about 4 mm. The load continues to increase in a nonlinear manner, reaching a maximum value of 65 N, corresponding to a displacement of about 4.5 mm, followed by a gradual decrease. Figure 14b shows the test results for the specimen with CNT film added between the layers. The peak load is reached at the end of the linear section, about 45 N, corresponding to a displacement of about 3 mm, and the load decreases rapidly after reaching the peak, indicating that the unstable expansion of the crack occurs. Compared with the untoughened specimens, the peak load of the specimens with CNT film added is reduced by 33% and decreases rapidly after the peak, indicating that the addition of CNT film made the ability of the material to resist delamination significantly weakened. Figure 15 shows the load-displacement curves of the specimens prepared from carbon fiber dry cloth by the VARTM process. As seen from the test results of the untoughened specimen shown in Figure 15a, the load peaks at the end of the linear section and then decreases. For the toughened material, the peak load is higher than that of the untoughened material. The load continues to peak at a non-linear increase after the linear section and then decreases gradually.

**Figure 16:**
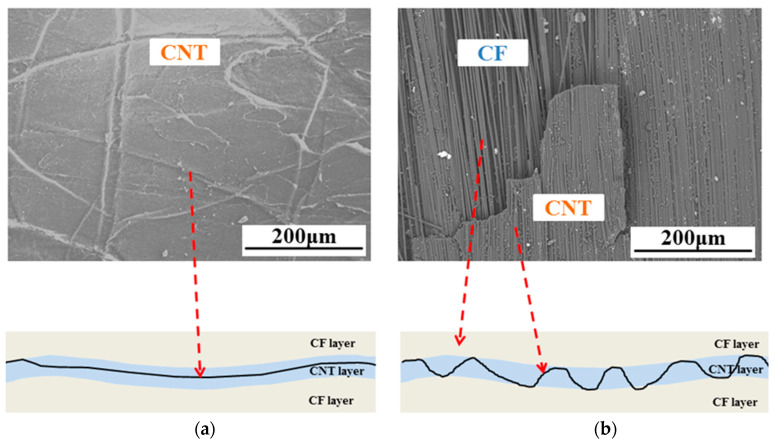
Cross sectional morphology of materials obtained by two preparation methods after mode I fracture. (**a**) Prepreg curing by hot press. (**b**) Carbon fiber curing by VARTM.

The comparison revealed a significant difference in the fracture properties of the materials prepared by the two methods after the addition of CNT films between the layers. The peak load of the toughened material prepared by prepreg via hot press tank was lower than that of the toughened material prepared by carbon fiber via VARTM process. In addition, the load of the toughened material prepared by prepreg decreases faster after reaching the peak, while the load of the toughened material prepared by carbon fiber dry cloth decreases more slowly. The above results indicate that the interlaminar crack extension of the CNT film-toughened composites prepared using the VARTM process are more stable. The fracture surfaces of the specimens are observed by scanning electron microscopy. From Figure 16a, it can be seen that the fracture surface of the prepreg specimen is covered with CNT film. The fracture surface is relatively flat and no carbon fiber is seen, which indicates that the crack is expanding inside the CNT film. Part of the CNT film and the carbon fiber underneath it exist on the fracture surface of the specimen prepared by VARTM curing method using carbon fiber dry cloth (Figure 16b), which indicates that the crack is zigzagging inside the CNT-toughened region stable extension. This is due to the fact that sufficient resin is available when utilizing the VARTM process so that the CNT film is fully impregnated. In comparison, when curing the carbon fiber prepreg directly using the hot press pot process, the limited resin content does not allow sufficient impregnation of the CNT film, and therefore delamination occurs within the film.

**Figure 17:**
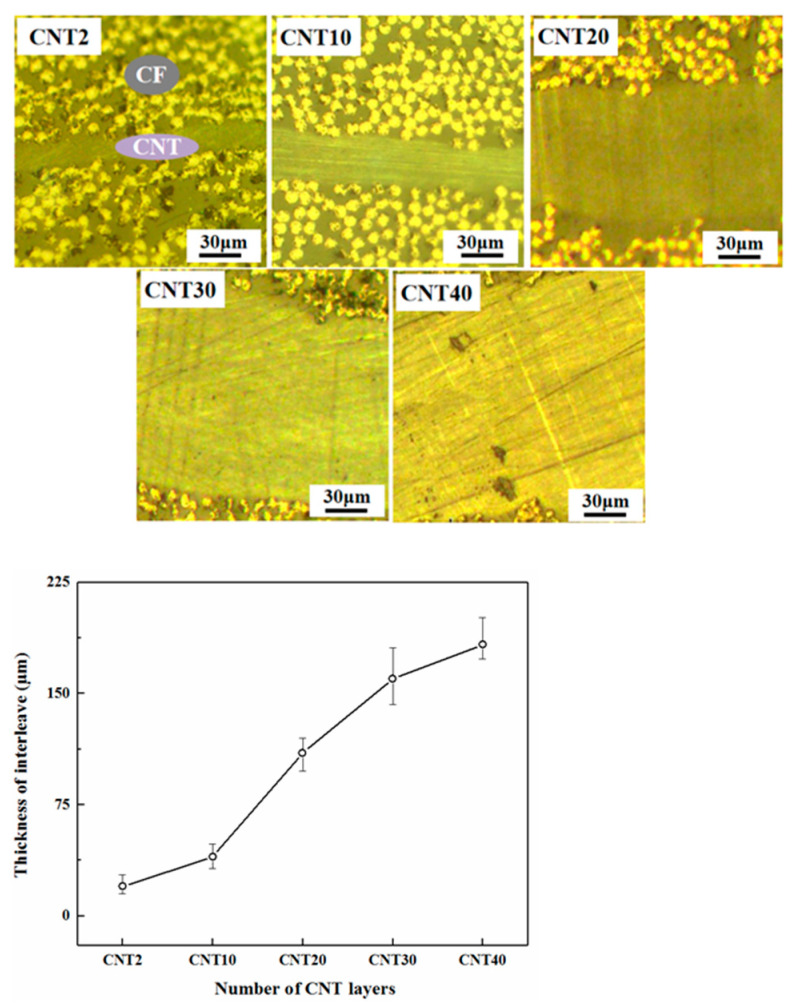
Variation of the thickness of interlaminar CNT-toughened area with the number of CNT film layers.

Layers of CNT films (n = 2, 10, 20, 30 and 40) are deposited to investigate the effect of the number of CNT layers on the thickness of the modified toughened zone. After the specimens are prepared, the interlayer thickness is measured by digital microscopy. The results are shown in Figure 17, which shows that the interlayer thickness increased with the increase of the number of CNT layers in a linear trend.

## 4. Conclusions

The interlaminar mode I fracture behaviors of CNT-modified carbon fiber composites are compared with those of unmodified materials, and the compressive mechanical properties of the two materials in both in-plane and out-of-plane directions at different strain rates. The following conclusions are obtained: (1) The compressive strength of CNT/CF/EP in the in-plane direction increased by 26% under quasi-static conditions, while the compressive strength in the out-of-plane direction increased insignificantly by about 4%, and the compressive strength in the out-of-plane direction is higher than that in the in-plane direction. (2) The compression strength of CNT/CF/EP increased by about 9% and energy absorption increased by about 14% when compressed dynamically in the out-of-plane direction, which is mainly due to the hindering effect of CNT on the generation and expansion of interlaminar cracks. The compression performance of CNT/CF/EP does not improve when compressed dynamically in the in-plane direction. The CNT-dominated interlaminar delamination and fiber crushing are the main failure modes, which cause the internal delamination of the film due to the limited resin content in the prepreg. It is the reason that the compression performance of CNT/CF/EP does not improve. (3) When using the VARTM process, the interlayer mode I fracture performance is better because the resin content inside the material is sufficient to avoid delamination inside the CNT film. (4) As the number of CNT layers increases, the thickness of the interlayer toughening zone thickness increases accordingly.

## Figures and Tables

**Table 1:** Compressive properties of CF/EPCNT/CF/EP.

Specimen	Strain Rate/s^−1^	Compression Strength/MPa
Out-of-plane		
CF/EP	10^−3^	670.8 ± 5
CF/EP	1000	843.3 ± 20
CF/EP	2500	799.5 ± 10
CNT/CF/EP	10^−3^	687.3 ± 4
CNT/CF/EP	1000	881.1 ± 9
CNT/CF/EP	2500	847.1 ± 15
In-plane		
CF/EP	10^−3^	321.0 ± 5
CF/EP	600	621.4 ± 5
CF/EP	1000	676.3 ± 12
CNT/CF/EP	10^−3^	405.8 ± 6
CNT/CF/EP	600	699.3 ± 10
CNT/CF/EP	1000	646.0 ± 16

## Data Availability

The raw data needed to reproduce these findings cannot be shared at this time, as the data will be used in ongoing research.
